# Supramolecular Complexes in Cell Death and Inflammation and Their Regulation by Autophagy

**DOI:** 10.3389/fcell.2019.00073

**Published:** 2019-05-03

**Authors:** Ian E. Gentle

**Affiliations:** Faculty of Medicine, Institute of Medical Microbiology and Hygiene, Medical Center, University of Freiburg, Freiburg im Breisgau, Germany

**Keywords:** inflammation, cell death, death domain, RHIM, supramolecular complexes, innate immunity, autophagy, cargo receptors

## Abstract

Signaling activation is a tightly regulated process involving myriad posttranslational modifications such as phosphorylation/dephosphorylation, ubiquitylation/deubiquitylation, proteolytical cleavage events as well as translocation of proteins to new compartments within the cell. In addition to each of these events potentially regulating individual proteins, the assembly of very large supramolecular complexes has emerged as a common theme in signal transduction and is now known to regulate many signaling events. This is particularly evident in pathways regulating both inflammation and cell death/survival. Regulation of the assembly and silencing of these complexes plays important roles in immune signaling and inflammation and the fate of cells to either die or survive. Here we will give a summary of some of the better studied supramolecular complexes involved in inflammation and cell death, particularly with a focus on diseases caused by their autoactivation and the role autophagy either plays or may be playing in their regulation.

## Structural Elements for Supramolecular Signaling Complexes

In order to assemble supramolecular signaling complexes in a tightly regulated fashion, certain protein–protein interaction domains and motifs have evolved. By using shared interaction mechanisms, these structures can assemble many subunits of differing function in a rapid and modular fashion to facilitate signal transduction. There are likely many other examples of proteins and domains that fall into this category, but in this review we have chosen to focus on the following structural elements due to their significant representation in the pathways regulating cell death and inflammation.

### Death Domain Family

**FIGURE 1:**
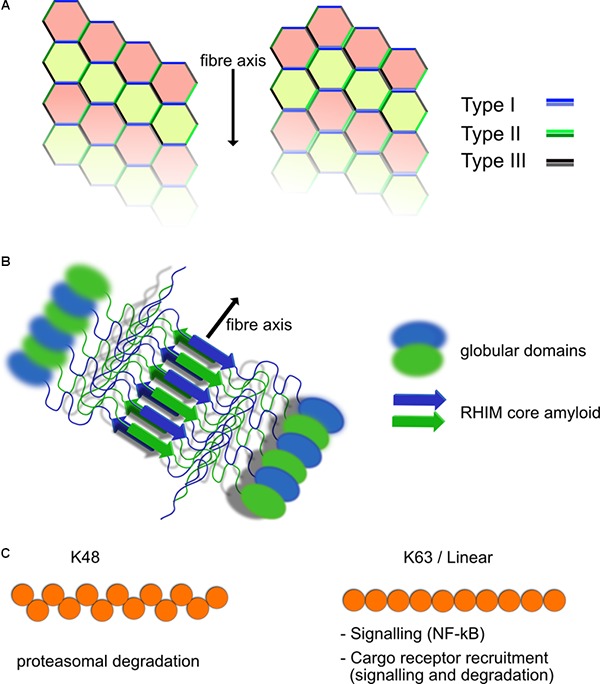
Structural elements of death domain family, RHIM and ubiquitin scaffolds. **(A)** Death domain family interaction can form fibrillar structures. Death domain family members interact with themselves through three different interaction modes known as Types I–III, depending on the surfaces of the domain that interact. Shown are two possible helical fibrillar assemblies using differing combinations of interfaces. Multiple forms of this type of interaction have been identified, allowing for modular assembly of different complexes. **(B)** RHIM–RHIM scaffolds form amyloid fibrils. Shown is a fibril of two proteins containing a RHIM and globular domains. The fibril is formed by two parallel beta amyloid sheets coming together. This brings the globular domains into close proximity for interaction and potential activation such as kinase domains of RIPK1/3. RHIM fibrils can be mixed or homogeneous (RIPK1–RIPK3 or RIPK3–RIPK3 fibers form example). **(C)** Polyubiquitin chains have different functional roles. Shown are K48 and K63/linear ubiquitin chains. The structural layout of the individual chains is different resulting in recruitment of different ubiquitin binding proteins. K63 and linear ubiquitin chains are similar in their layout, although still functionally distinct. K48 chains are predominantly used for proteasomal degradation, whereas K63 and linear ubiquitin chains are used for recruitment of NF-κB activating complexes such as TAB/TAK and IKK as well as linking to autophagic cargo receptors among other functions.

The death domain containing protein family is involved in numerous aspects of cell signaling and fate and contains several subfamilies including Caspase Activation and Recruitment Domain (CARD), Death Effector Domain (DED), Pyrin Domain (PYD), and the Death domain (DD) itself (Nanson et al., 2018). These domains share structural and sequence similarity but they show specificity with their interactions and tend to interact within each subfamily specifically, CARD–CARD or DD–DD interactions for example. Each member of the family mediates protein–protein interactions and seem to typically form Helical assemblies, some of which are fibrillar in nature such as ASC in inflammasomes (Lu et al., 2014; Li et al., 2018). Members of the Death Domain family interact through three distinct interaction types known as Types I–III (Figure 1A). Depending on the arrangement and combination of the different interaction interfaces between DD family proteins, different structural arrangements can be generated (Figure 1). Clustering of death domain family proteins leads to recruitment of effector proteins including caspases but also ubiquitin ligases and deubiquitinases (DUBs), kinases and other regulatory proteins involved in signal transduction. Through the ordered clustering provided by death domain structures, proteins requiring oligomerization such as caspases are locally concentrated to stimulate interaction and subsequent activation. Interruption of these Death Domain family interactions blocks function suggesting that their assembly is required for activity (Park et al., 2007).

### Receptor Homotypic Interaction Motif (RHIM)

Another structural element contained in supramolecular complexes discussed in this review and which has been shown to play a crucial role in inflammatory and cell death signaling is the Receptor Homotypic Interaction Motif (RHIM) (Sun et al., 2002). RHIMs are relatively short motifs characterized by a core sequence that has the property of folding into a highly stable amyloid structure (Pham et al., 2019) (Figure 1B). Proteins that include RHIM motifs interact with each other via these RHIM–RHIM interaction and include RIPK1, RIPK3, TRIF, DAI/ZBP1. Each of these may potentially interact with the other, however, it is not clear that all combinations are seen under normal situations in the cell (Pham et al., 2018). Mutation of RHIM motifs in RIPK3 for example is enough to abolish its activity suggesting that its role in polymerizing partners together is not separable from other functions it may have (Li et al., 2012). Large structures have been demonstrated for both RIPK1 and 3 as well as TRIF, which are dependent on RHIM interactions (Li et al., 2012; Gentle et al., 2017; Samie et al., 2018). In the case of TRIF, we have shown that these structures are fibrillar complexes that contain at least RIPK1 as well and probably also RIPK3 and can activate caspase-8 and other signaling outcomes of TRIF mediated signaling (Gentle et al., 2017). RHIM–RHIM interaction also provide important scaffolds for recognition of viral infection and subsequent cell death, such as is observed in influenza A virus infections triggering a DAI/ZBP1 and RIPK3 dependent cell death (Thapa et al., 2016). RHIM containing proteins are often recruited to complexes formed through Death Domain family interactions and RIPK1 indeed, has both a RHIM and Death Domain to promote this. How the architecture of such supramolecular complexes formed through RHIM and Death Domain scaffolds looks is still an unknown question, but what is clear is that loss of either of them can drastically alter signaling outcomes.

### Ubiquitin

A common component and key player in the regulation of supramolecular signaling complexes is ubiquitin. In all complexes described in this review, polyubiquitin chains are attached to one or more of the subunits. A common signal activating function of ubiquitin chains in these complexes is to recruit kinase complexes IKK and TAB/TAK to activate NF-κB. However, the same K63 chains are also involved in the eventual silencing of the signaling complexes (see later). Other linkage specific chains are also present including K48 and Met1. Met1 which is added by the Linear UBiquitin chain Assembly Complex (LUBAC) is also important for NF-κB activation through recruitment of IKK complexes in a similar fashion to and in cooperation with K63 chains (Hrdinka and Gyrd-Hansen, 2017). K48 chains are typically associated with proteasomal turnover and are also important for regulation of signals such as from TNFR1 through proteasomal degradation of RIPK1 (Grice and Nathan, 2016; Annibaldi et al., 2018). Depending on the substrate and site of attachment, these ubiquitin chains may also regulate interactions with partner proteins and thus activity, for example recruitment of the NF-kB inducing kinases. The ubiquitin network within a signaling complex is a dynamic one, with the competing actions of ligases and deubiquitinases (DUBs) modifying the overall outcome of the signaling event, regardless of the receptor complex in question. This is a very complicated system and beyond the scope of this review to cover in any depth and has been reviewed thoroughly (Swatek and Komander, 2016). We will focus instead mostly on the role ubiquitin plays regulating recruitment to the autophagy machinery and activation of NF-κB.

## Switching Off Large Signallng Platforms

Assembly for supramolecular complexes such as those discussed throughout this review, presents a potential problem in terms of switching the signal off. Given the damaging outcome of overactivation of inflammatory or cell death promoting complexes, these structures need to be silenced before they can lead to cellular or tissue damage and disease. While this may in part be regulated by post translational modifications such as (de)ubiquitination, and (de)phosphorylation the likely stability and energetic requirement to break apart complexes such as RHIM or CARD mediated fibrils make this an unlikely mechanism to completely explain disassembly and inactivation of the fully assembled complexes. Degradation of subunits of these complexes by the proteasome may also play some role, however, the size of the complexes discussed in this review make it unlikely that proteasomal degradation of the assembled complexes occurs. Access to the proteasome typically requires unfolding of the substrate to allow it to fit within the catalytic barrel (Collins and Goldberg, 2017). Individually unfolding proteins assembled into supramolecular complexes, while perhaps possible, is likely an inefficient way to silence the activity of these complexes, however, is certainly a relevant regulatory mechanism for preventing their assembly through degrading members of the complexes before they can be recruited. Autophagy represents an existing mechanism capable of dealing with very large protein aggregates.

**FIGURE 2:**
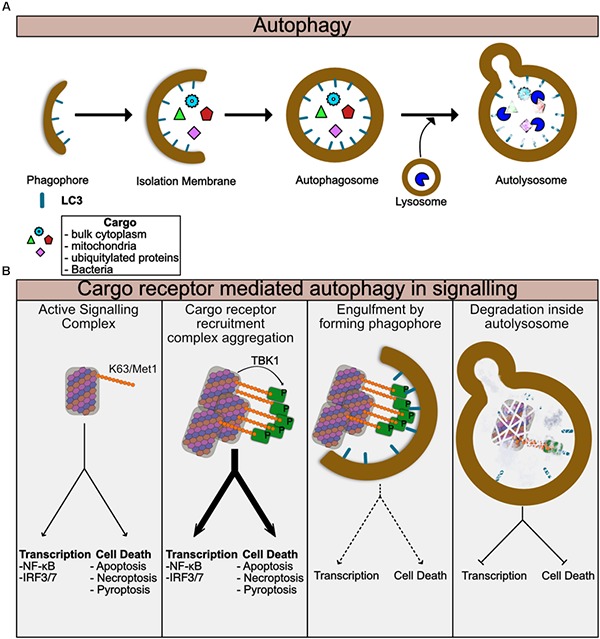
Autophagy regulates turnover of Supramolecular complexes. **(A)** Summary of general autophagic process. The autophagic machinery including LC3 is recruited to the donour membrane and forms the phagophore. This then extends to form the isolation membrane which begins to engulf cytoplasmic or ubiquitylated contents. Eventually the enclosed autophagosome is formed which then can fuse with lysosomes to form the autolysosome. Lysosomal enzymes then degrade the contents of the autolysosome. **(B)** Summary of specific autophagy mediated by cargo receptors. Assembled supramolecular signaling complexes begin their signaling response. Cargo receptors such as p62 are recruited via K63 ubiquitin chains on the complex. TBK1 which has been activated and recruited to the complex phosphorylates the cargo receptor to enhance its recruitment. At this stage the signal from the complex may be amplified due to clustering of multiple complexes together or perhaps further enhancement of the localized proximity of kinases, caspases, and ubiquitin ligases for example. As the cargo:cargo-receptor complexes are engulfed by the forming autophagosome, signaling will be reduced. Finally, degradation of the complex within the autolysosome completes the cycle and the complex is destroyed.

Autophagy is at its heart a cellular recycling mechanism to provide energy in times of nutrient stress, but has been adapted in multicellular organisms to also regulate multiple aspects of cellular biology. The canonical pathway also known as Macroautophagy creates so-called autophagosomes, membranous vesicles, which engulf random parts of the cytosol and organelles (Zhao and Zhang, 2018) (Figure 2A). These autophagosomes fuse with lysosomes and degrade the contents which are then recycled for use in energy catabolism or for synthesis of new molecules. More specific forms of autophagy have evolved to degrade particular targets including mitochondria (mitophagy), intracellular bacteria and viruses (Xenophagy) and also aggregated proteins (aggrephagy) among others. In each of these targeted autophagic pathways, the target is ubiquitylated, typically by K63 ubiquitin chains (Sharma et al., 2018). These ubiquitylated targets are detected by so-called cargo receptors through binding to ubiquitin chains. This process is now known to be regulated by phosphorylation by TBK1, which will be discussed later in the review more specifically (Figure 2A). The receptors then recruit LC3, a component of the forming autophagophores causing engulfment of the cargo receptor bound target in autophagosomes (Figure 2B). The cargo containing autophagosomes can then fuse with lysosomes and degrade the contents (Figure 2B). Thus, autophagy is ideal for switching off signals from supramolecular signaling complexes. It is of note that many of the signaling pathways using supramolecular complexes such as toll like receptors (TLR) and TNF Receptor family signals, also induce autophagy, thus stimulating the pathways involved in their silencing (Lee et al., 2018; Liu Y. et al., 2018). Examples of specific autophagy of signaling molecules are given throughout this review.

## Supramolecular Signaling Complexes in Cell Death and Inflammation

### TNF Family Receptors

**FIGURE 3:**
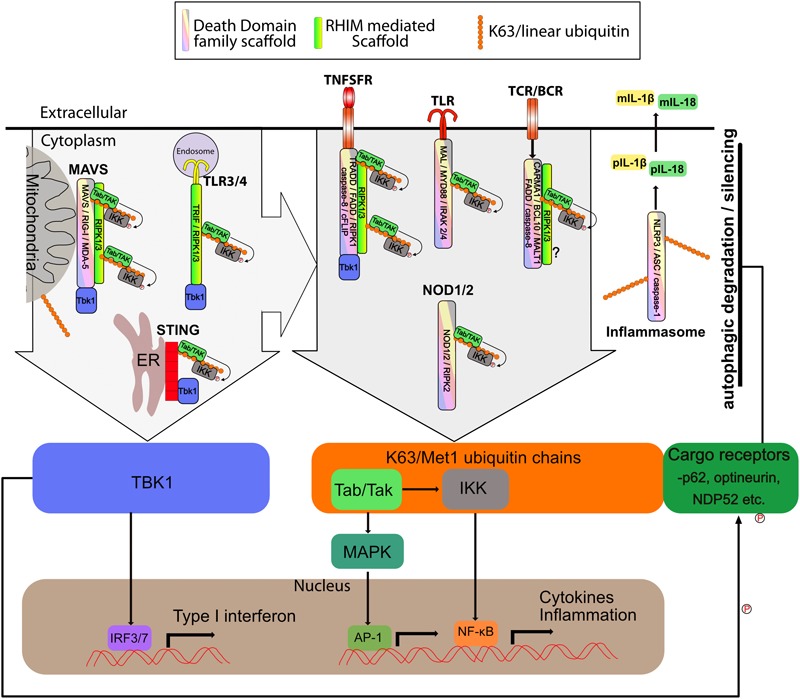
Supramolecular signaling complexes in inflammation share common scaffolds and signaling pathways. The supramolecular signaling complexes formed by TLR, TNFSR, NOD1/2, STING, MAVS, Carma, BCL-10, MALT1 (CBM) and inflammasome complexes are indicated. Shown are the core scaffolds formed through death domain family interactions as well as RHIM interactions. STING contains no death domain family member or RHIM. Specific proteins known to interact through death domain interactions and RHIM interaction are indicated within each complex. Recruitment of TBK1 and/or K63/linear ubiquitin is indicated. TBK1 recruitment activates IRF3 to induce interferon responses. K63/linear ubiquitin recruits the TAB/TAK and IKK complexes which result in activation of NF-kB and Map kinase transcriptional responses. Also shown is linear/K63 ubiquitination of ASC of inflammasomes which can promote their assembly and activation or degradation. Complexes and organelles such as mitochondria that are k63 ubiquitylated recruit autophagy cargo receptors such as p62. This leads ultimately to degradation and silencing of the complexes. This is enhanced by TBK1 mediated phosphorylation of the cargo receptors. Other scaffolds such as those mediated via TRAF proteins are also present, but for simplicity have been omitted from the figure. DD family and RHIM scaffold are not meant to be to scale or reflect the actual organization of the scaffold, but simply indicate that each of these scaffolds are present.

**FIGURE 4:**
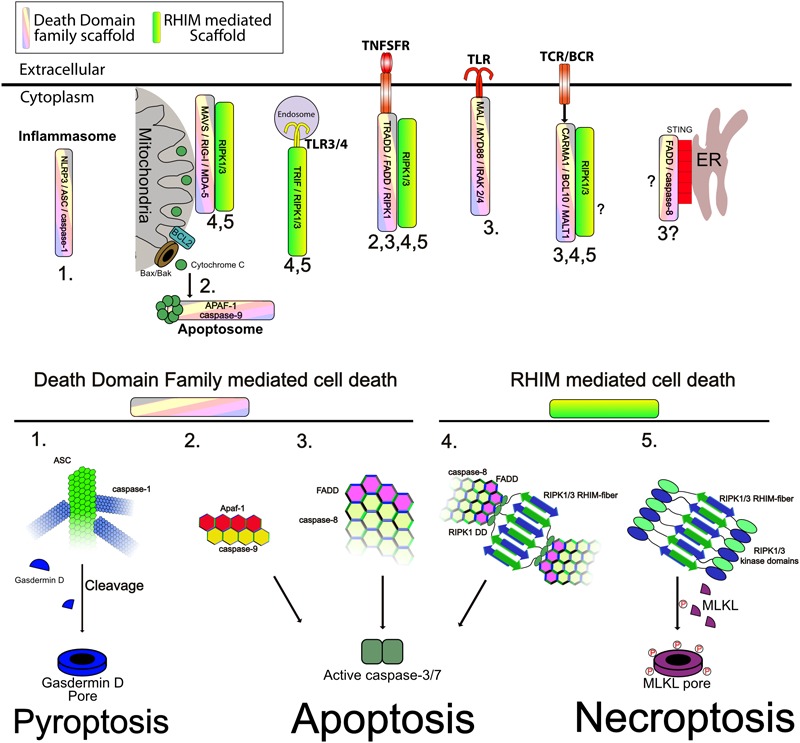
Supramolecular signaling complexes can activate cell death. **(Upper)** The indicated complexes and the respective scaffolds recruited are indicated. Numbers correspond to the modes of cell death shown in the lower panel ad indicate the modes shown to be triggered by the respective complexes. **(Lower)** Different modes of cell death and the role of the respective scaffold in activating caspases, or RIP kinases is shown. (1) Inflammasomes assemble a core filament of ASC which in turn recruits filaments of caspase-1, to cluster it and trigger its activation. Active caspase-1 then cleaves Gasdermin D to allow the cleaved form to assemble into multimeric pores in the plasma membrane, causing pyroptosis. (2) Release of cytochrome C through Bax/Bak pores on the mitochondrial outer membrane triggers formation of the apoptosome. The apoptosome consists of a core of APAF1 and caspase-9 interacting through CARD–CARD interactions as indicated. This allows activation of caspase-9. Caspase-9 then cleaves and activates caspase-3/7 to trigger apoptosis. (3) Caspase-8 clustering onto FADD causes its autoactivation. Clustering is mediated through DED–DED interactions between FADD and caspase-8 and caspase-8 itself (not shown is cFLIP which can commingle with caspase-8 to regulate its activation). Active caspase-8 can cleave caspase-3/7 and trigger apoptosis. This scaffold can be activated by numerous receptors as indicated. (4) Recruitment and activation of RIPK1/3 through RHIM–RHIM mediated amyloid fibers can trigger FADD recruitment and clustering through the DD of RIPK1. Clustered FADD recruits caspase-8, probably causing similar aggregation as shown in mode 3. Active caspase-8 cleaves caspase-3/7 to trigger apoptosis. (5) The kinase domains of RIPK1 and RIPK3 are also brought into close proximity by RHIM fibers. Normally, the capase-8 indicated in mode 4 will inactivate these through cleavage of their kinase domains, however, when no caspase-8 activity is present, as is shown, RIPK3 is activated and phosphorylates MLKL. Phosphorylated MLKL assembles into pore complexes in the plasma membrane, causing necroptosis.

Much of the best studied signaling complexes are in the TNF Super Family (TNFSFR), including TNFR1, FAS, TRAIL among others. Each of these receptors ultimately leads to activation of caspase-8 as well as activating transcriptional programs, particularly NF-κB. TNFSFR use death domain family interactions to recruit both scaffolding proteins such as TRADD and FADD, as well as effector proteins such as Caspase-8/10, and RIPK1, although many of the effector proteins also exhibit some scaffolding function, independent of their catalytic activities. Using TNFR1 as a well-studied example, TNFR1 recruits the adapter TRADD, which in turn recruits both RIPK1 and/or FADD. Recruitment of TRAF2/5 along with cIAP1/2 triggers k63 linked ubiquitylation of RIPK1 as well as other components. These ubiquitin chains recruit IKK as well as the TAB/TAK complexes and the linear ubiquitin chain assembly complex (LUBAC) which further adds linear ubiquitin chains. The IKK and TAB/TAK complexes then both activate NF-κB as well as phosphorylate RIPK1 to prevent its activation (Figure 3). This results in upregulation of inflammatory cytokines and cell survival. Loss of RIPK1 ubiquitylation, and thus recruitment of the IKK and TAB/TAK complexes, results in cell death via apoptosis when the so-called complex-II containing RIPK1-FADD-caspase-8 separates from the receptor and activates cytosolic caspase-8 leading to apoptosis (Figure 4) (Vince et al., 2007; Dondelinger et al., 2013, 2015, 2017; Jaco et al., 2017; Menon et al., 2017; Annibaldi et al., 2018). This cytosolic amplification of complex-II formation is likely also mediated by supramolecular assembly of the complex-II through DD and RHIM interactions. Structural models have been developed for the assembly of FADD and Caspase-8 into fibrillar complexes in response to TRAIL ligand (Dickens et al., 2012) or FasL (Fu et al., 2016). While these models differ in their assembly, the principle remains that large elongated networks of Caspase-8 are assembled to trigger its activation through proximity (Figure 4). It seems likely that RIPK1 dependent Caspase-8 activation in the cytosol via Complex-II follows a similar mechanism. To date no auto-activating mutants of FADD or caspase-8 have been identified in disease, this is likely due to their propensity to trigger apoptosis, however, somatic loss of function mutants or repression of expression of FADD and caspase-8 are associated with numerous cancers, and seem to drive NF-κB (Teitz et al., 2000; Shin et al., 2002; Kim et al., 2003; Tourneur et al., 2004; Soung et al., 2005; Ando et al., 2013). This speaks to the role that supramolecular complex assembly has in regulating the complicated network of signaling molecules that are recruited and that loss of function of one, can lead to hyperactivation of another or vice versa.

In the case of TNF family receptors, one important aspect of their activity is their endocytosis. Endocytosis is required for apoptosis from TNFR1 for example (Micheau and Tschopp, 2003; Schütze et al., 2008). Endocytosed receptors are thought to be able to either traffic back to the plasma membrane or are packaged into multivesicular bodies (MVB) that ultimately fuse with lysosomes and are degraded. In this regard, endocytosis and trafficking via vesicles acts as an in-built silencing mechanism for TNFSFR proteins. However, targeted autophagy was recently identified to regulate the levels of fn14 suggesting that autophagic degradation may also occur in other TNFSFR too (Winer et al., 2018). Supporting this, optineurin (a homolog of NEMO and a functioning autophagic cargo receptor) blocks TNF induced NF-κB by binding ubiquitylated RIPK1 (Zhu et al., 2007). While not shown in this study, this likely also results in degradation of RIPK1/3 containing complexes. More recently, optineurin, mutations of which are associated with amyotrophic lateral sclerosis (ALS), was shown to trigger degradation of RIPK1 and its loss lead to axonal degeneration (Ito et al., 2016). Optineurin loss also sensitized L929 cells to necroptosis induction supporting its role in targeting RIPK1 and RIPK3 for autophagic degradation to prevent their accumulation and activation (Ito et al., 2016). Optineurin loss has also been shown to sensitize to TNF induced caspase-8 activation (Nakazawa et al., 2016), supporting it as having a negative regulation of TNFR1 signaling.

### Toll Like Receptors (TLRs)

Toll Like receptors, like TNFSFR, also signal through large complexes which may promote both inflammation and cell death. TLR’s, use specific adapter proteins to recruit signaling complexes to the receptor. More specifically, TLRs that signal through the adaptor TRIF/TICAM1 are able to recruit RIPK1 and through RIPK1 activate caspase-8 and or RIPK3 to trigger apoptosis or necroptosis (Kaiser and Offermann, 2005; Weber et al., 2010; Kaiser et al., 2013) (Figure 3, 4) TRIF is recruited to both TLR3, where it is the sole adapter, and TLR4, which also uses MYD88 like all other TLR’s (Hoebe et al., 2003; Oshiumi et al., 2003). Normally, TRIF mediated signaling results in assembly of K63 and linear ubiquitin chains that recruit the TAB/TAK and IKK complexes to trigger NF-κB (Figure 3) (Shim et al., 2005; Zinngrebe et al., 2016). TRIF also activates IRF3/7 mediated interferon responses via TBK1 activation (Fitzgerald et al., 2003; Hemmi et al., 2004) (Figure 3). In either case TRIF signaling complexes are restricted to endosomes (Figure 3, 4). Myd88 dependent signaling occurs through formation of the myddosome (Motshwene et al., 2009). The myddosome is a helical complex consisting of Myd88, IRAK4, and IRAK1/2 stacked together through Death Domain interactions in a manner similar to that shown in Figure 1A (Lin et al., 2010). This platform recruits TRAF6, leading to IKK activation and NF-κB (Häcker et al., 2006) (Figure 3). While some evidence exists for apoptosis induced through MYD88 from TLR2 through recruitment of FADD and caspase-8, this seems to be the exception rather than the rule (Figure 4) (Aliprantis et al., 2000).

In either case, both MYDD88 and TRIF adapters have been shown to form supramolecular complexes which are required for several aspects of their signaling activity (Funami et al., 2007; Tatematsu et al., 2010; Guven-Maiorov et al., 2015; Gentle et al., 2017; Samie et al., 2018). Indeed Mutations in Myd88 that are associated with Lymphoma trigger auto-assembly of the TIR domain and the myddosome without receptor ligation (Avbelj et al., 2014). To date no auto-activating mutations of TRIF have been identified, possibly due to its capacity to trigger cell death when overactive. Mutations in FADD that are associated with cancers have also been shown to interfere with the association of FADD with MYD88, which may in turn promote myddosome assembly (Guven-Maiorov et al., 2015).

A number of studies have now associated regulation of TLR signaling via autophagy. Specifically Myd88 was reported to be recruited to p62 and Histone deacetylase 6 (HDAC6) positive structures upon TLR4 stimulation (Into et al., 2010; Fujita et al., 2011). p62 acts as a cargo receptor for selective autophagy and HDAC6 is thought to help traffic the ubiquitylated complexes to bring them together and promote fusion of the autophagosomes with lysosomes (Kawaguchi et al., 2003; Lee et al., 2010). Loss of p62 or HDAC6 resulted in enhanced JNK, p38, and ERK signaling in response to TLR ligands, but appeared to have little effect on NF-κB. While it was not specifically demonstrated in these studies, the enhanced signaling, albeit through specific pathways, is suggestive of the myddosome being targeted by autophagy, loss of which prevents the signals being dampened.

TRIF can form large fibrillar complexes that have been variably reported to interact with the autophagy cargo receptors p62, Tax1BP1, and NDP52 to target them for degradation (Inomata et al., 2012; Gentle et al., 2017; Yang et al., 2017; Samie et al., 2018). Our own study showed that if autophagy is inhibited, not only did the transcriptional activity of TRIF signaling become enhanced resulting in increased cytokine production, but also the cell death inducing activity, with enhanced caspase-8 activation being detected upon TLR3 stimulation of melanoma cells (Gentle et al., 2017). Similar results were shown in the other studies indicating enhanced IFN production in response to polyI:C or LPS when autophagy was inhibited (Samie et al., 2018). TRIF can also interact with the ubiquitin like protein ubiquitilin1 driving association with autophagosomes and degradation of TRIF (Biswas et al., 2011). Loss of ubiquitilin1 leads to excessive type I interferon responses from TLR4 and TLR3 (Biswas et al., 2011). Additionally TRIF can also induce autophagy as is the case with most of the complexes discussed here (Gentle et al., 2017).

As with TNFRSF receptors that become endocytosed, TLRs, in part at least, are associated with endosomes, an aspect which is essential for their activity (Petes et al., 2017). In addition to the autophagic regulation discussed above, a similar fate probably awaits activated TLRs that are present on endosomes such as TLR3 and TLR4 containing TRIF complexes (Wang et al., 2007). What ultimately happens to plasma membrane bound TLRs after assembly of myddosomes is unclear. They may also be trafficked via general endocytosis or they may, as is the case with TNFR1 complex II dissociate from the receptors themselves and then become targeted by autophagy separately.

### Inflammasomes

Inflammasomes are another example of a death domain based supramolecular complex that has potent inflammatory signaling activity but can also trigger death of cells. There are a number of inflammasomes, however, the majority share a core structure, with a receptor subunit such as NLRP3 activating and recruiting Apoptosis-associated speck-like protein containing a CARD (ASC), which is able to then polymerize into a helical fibrillar structure that recruits Caspase-1 (Lu et al., 2014; Li et al., 2018). This brings caspase-1 into close proximity with other caspase-1 molecules, triggering auto-processing and activation of the zymogen into its functional form. Caspase-1 then cleaves the cytokines IL-1β and IL-18 to trigger further inflammation, but can also cleave substrates including Gasdermin D which then forms pores in the plasma membrane leading to a type of cell death called Pyroptosis (Martinon et al., 2002; He et al., 2015; Shi et al., 2015; Liu et al., 2016) (Figure 3, 4). Pyroptotic death is additionally thought to release danger associated molecular patterns (DAMPS) that trigger inflammation in and of themselves, thus promoting an inflammatory environment (Frank and Vince, 2018). Auto-activating mutations in inflammasome components are known and cause diseases such as Familial Mediterranean Fever (FMF) and cryopyrin-associated periodic syndrome (CAPS) (Cordero et al., 2018). These mutations typically lead to auto-assembly of the inflammasome and lead to severe inflammatory pathologies due to the increased secretion of IL-1β and II-18 and pyroptosis (Cordero et al., 2018). Efficiently regulating the signal strength and length of signaling through inflammasomes is therefore essential. Autophagy plays a number of roles in regulating inflammasomes. Both the AIM2 and NLRP3 inflammasomes can be recruited to p62 and engulfed by autophagosomes and later associate with lysosomes for degradation (Shi et al., 2012). Blocking autophagy enhances inflammasome activity and stimulating autophagy reduces it (Shi et al., 2012). Loss of autophagy is also associated with enhanced NLRP3 activation and IL-1β secretion (Saitoh et al., 2008), although the mechanism behind this activation is not yet clear. A number of studies have suggested that mitochondrial defects, possibly through insufficient mitophagy, may promote inflammasome activation through excessive ROS production or possibly release of ligands such as mtDNA (Ip et al., 2017). Stimuli that induce inflammasome activation also induce autophagy (Shi et al., 2012), supporting its role as a negative feedback system for these complexes.

### STING

STING is activated by cyclic dinucleotides (cGAMP) produced by cGAS upon detection of cytoplasmic DNA (Ablasser et al., 2013). cGAMP binding causes STING to assemble into a large ER associated complex that leads to activation of NF-κB through IKK activation upon recruitment to K63 and linear polyubiquitin chains, but also to IRF3 through recruitment and activation of TBK1 (Abe and Barber, 2014) (Figure 3).

STING activation can also induce apoptosis in T cells and B-cell lymphomas although the mechanism is still unclear (Tang et al., 2016; Gulen et al., 2017; Larkin et al., 2017). Auto activating mutations of STING cause STING-associated vasculopathy with onset in infancy (SAVI), which among other problems results in T-cell cytopenia. This is independent of IRF-3 and could be due to cell death of developing or mature T-cells based on mouse knock-in models (Warner et al., 2017; Wu and Yan, 2018). Autoactivating mutations of STING trigger dimerization in the absence of ligand, allowing the signaling complex to form (Konno et al., 2018). FADD deficiency can block IFN activation in STING activated cells, suggesting that STING requires FADD and providing a possible mechanism for how STING may induce cell death through apoptosis (Ishikawa and Barber, 2008) (Figure 4). Additionally, STING can also cause necroptotic cell death indirectly through induction of TNF and interferon in macrophages and dendritic cells (Brault et al., 2018).

STING has also recently been identified as an autophagic substrate. Upon activation it binds to p62 and is targeted for degradation through autophagy (Liu D. et al., 2018; Prabakaran et al., 2018). STING itself can also act as an autophagy cargo receptor through an LIR motif to directly recruit LC3 and form autophagosomes around it after activation (Liu D. et al., 2018), although why it should need its own LIR when p62 is also recruited remains to be seen. Loss of p62 causes strongly enhanced IFN stimulated gene expression in response to cytosolic DNA indicating that STING activity is much higher. STING also induces autophagy in order to stimulate its own degradation (Liu D. et al., 2018) and the autophagy inducing kinase ULK1 also phosphorylates activated STING to negatively regulate its function (Konno et al., 2013). STING directly promotes the lipidation of LC3 at the ER to promote autophagosome production (Gui et al., 2019). This function of STING appears to have evolved before interferon activation as demonstrated by a lack of TBK1 and IRF activation by both Xenopus and sea anemone STING homologs, and is important for clearance of HSV-1 (Gui et al., 2019). Together, these studies support the picture of autophagy regulating signaling platforms and vice versa.

### Mitochondrial Antiviral-Signaling Protein (MAVS)

Mitochondrial antiviral-signaling protein (MAVS) is a signaling scaffold for detection of viral RNA products. The receptors RIG-I and MDA5 bind to viral dsRNA molecules and then oligomerize (Berke et al., 2012; Jiang et al., 2012). Exposure of their CARD domains allows them to bind to the card domains of MAVS on the mitochondrial outer membrane whereby MAVS polymerizes into supramolecular fibrillar complexes to act as a scaffold for recruitment of IKK and TBK1 complexes for the activation of NF-κB and IRF3 (Figure 3) (Seth et al., 2005; Hou et al., 2011; Xu et al., 2014). Additionally FADD, RIPK1, RIPK3, and caspase-8 can be recruited to these complexes (Kawai et al., 2005; Downey et al., 2017). Infection with Semliki Forest virus induces apoptotic cell death through caspase-8 activation via the MAVS pathway highlighting that there can be a direct death inducing signal triggered by MAVS (Figure 4) (El Maadidi et al., 2014). Again, the MAVS complexes assemble through CARD–CARD interactions, and while no auto-activating mutations are known in humans for MAVS itself, gain of function mutations are found in both mda5 and RIG-I and are associated with type I interferonopathies such as Aicardi-Goutières syndrome (AGS) and Atypical Singleton-Merten syndrome (SMS) (Rice et al., 2014; Jang et al., 2015; Rutsch et al., 2015). These mutations can lead to enhanced fibril formation of the MAVS complex through an unknown mechanism that may be due to enhanced activation of the receptors themselves leading to excessive MAVS polymerization (Funabiki et al., 2014; Rice et al., 2014).

While MAVS appears to be an autophagy substrate it is perhaps a somewhat special case in this regard due to its location on the mitochondria. MAVS is reported to contain an LIR motif for direct recruitment of LC3 and MAVS activation and assembly into its large fibrillar state can stimulate mitophagy (Sun et al., 2016), thus removing MAVS along with the mitochondrial it is associated with. Additionally, MAVS can be degraded by autophagy through an NDP52 dependent mechanism regulated by the interferon response gene tetherin and loss of NDP52 gives an enhanced interferon response, albeit minor (Jin et al., 2017). The relatively minor effect that loss of NDP52 has on MAVS signaling, may be due to MAVS acting as its own cargo receptor and inducing autophagy/mitophagy. Clearly, however, MAVS adheres to the pattern shown for the other complexes so far discussed in using autophagy as a silencing mechanism.

### NOD2

NOD2 is another CARD containing PRR that recognizes bacterial muramyl dipeptide (MDP) (Girardin et al., 2003). Through its CARD domain NOD2 recruits RIPK2, which is then ubiquitylated by XIAP resulting in the recruitment of the TAB/TAK and IKK complexes and NF-κB activation through addition of linear ubiquitin chains via LUBAC (Figure 3) (Damgaard et al., 2012, 2013). Recently, RIPK2 was shown to also form filaments through CARD–CARD interactions and filament formation and scaffolding function is required for NOD2 activity (Hrdinka et al., 2018; Pellegrini et al., 2018). Interestingly, crosstalk between the NOD2 and MAVS pathway has been demonstrated during viral infection. ssRNA is recognized by NOD2 and it then interacts with MAVS triggering IRF3 activation through TBK1, although surprisingly, not through CARD–CARD interactions (Sabbah et al., 2009). Mutations in NOD2 are associated predominantly with Crohn’s Disease and result in a failure to induce NF-κB in response to ligand (Hampe et al., 2001; Hugot et al., 2001; Ogura et al., 2001; Bonen et al., 2003). However, there is some question as to whether this is the causative defect in Crohn’s Disease (Eckmann and Karin, 2005). Another disease thought to be caused by autoactivating mutations of NOD2 is Blau syndrome. Patients with Blau syndrome exhibit granulomatous dermatitis, arthritis, and uveitis (Kanazawa et al., 2005; Parkhouse et al., 2014) Although, again the autoactivation of NOD2 driving disease has also been questioned (Dugan et al., 2015). Given the fibril forming nature of NOD2-RIPK2 complexes, it seems likely that hyperactivation could result from aberrant assembly of RIPK2 fibrils, however, this has not been demonstrated yet.

NOD2 has a complex interaction with the autophagy machinery. As is the case with most of the complexes discussed here, NOD2 can induce autophagy. It is thought that this occurs through a RIPK2 kinase dependent mechanism (Homer et al., 2012). Additionally, NOD2 is also involved in the recruitment of autophagic machinery to sites of invading bacteria at the plasma membrane through interaction with Atg16. This targets the bacteria for degradation via xenophagy (Cooney et al., 2010; Homer et al., 2010, 2012; Travassos et al., 2010; Anand et al., 2011; Negroni et al., 2016). To date no degradation of NOD2 or its complexes have been shown via autophagy. NOD2 is thought to be a proteasomal substrate under conditions where HSP90 is blocked or its access to NOD2 is restricted (Normand et al., 2018). RIPK2, however, has been identified as interacting with p62, although autophagic degradation was not shown in this study (Park et al., 2013). The NOD2-RIPK2 fibril may therefore also be a target of autophagic degradation given this association and may play a role in dampening NOD driven inflammation.

### CBM Signalosome

In a similar manner to inflammasomes, the CARMA-BCL10-MALT1 (CBM) signaling complex consists of a core of BCL10 that recruits the paracaspase MALT-1. Different CARD containing adapters can recruit BCL10 to trigger its assembly including CARD9, -10, -11, and CARD14 (Gehring et al., 2018; Juilland and Thome, 2018). CARD11/CARMA1 is one of the best studied to date and is activated by TCR and BCR signaling. CARMA1 recruits BCL10 through CARD–CARD interactions, whereupon BCL10 forms filamentous helical structures in a manner similar to what has been described here for other CARD mediated structures such as inflammasomes (Qiao et al., 2013; David et al., 2018; Schlauderer et al., 2018). These filaments assemble in a star like formation radiating out from CARMA1 nucleation points (David et al., 2018). MALT1 is a paracaspase which requires recruitment to BCL10 for activation. TRAF6 is also recruited via interaction with MALT1 and NF-κB is subsequently activated through recruitment of IKK complexes to ubiquitin chains (Figure 3) (Sun et al., 2004). Recently it was shown that LUBAC is also required for NF-κB activation, although this may be due to a scaffolding role as catalytically inactive HOIP could also restore NF-κB signals in HOIP deficient cells (Dubois et al., 2014). FADD and caspase-8 are also recruited to the CBM complex and caspase-8 catalytic activity is required for effective activation of NF-κB by the CBM (Su et al., 2005). As loss of caspase-8 activity is a known trigger for necroptosis in stimulated T cells, these data also suggest that recruitment of FADD and caspase-8 are accompanied by RIPK1 and that this could be the route for activation of necroptosis in the absence of caspase-8 in stimulated lymphocytes (Figure 4).

Interestingly, mutations in CARD11 (Carma1) are causative of B cell expansion with NF-κB and T cell anergy (BENTA), an immunodeficiency caused by overactivation of the CBM complex. These mutations cause CARD11 to aggregate and recruit BCL10 and MALT1 into large complexes (Snow et al., 2012; Brohl et al., 2015). They are also often associated with diffuse large B cell lymphoma and other lymphomas (Lenz et al., 2008; Chan et al., 2013). CARMA3 has also been identified as a negative regulator of MAVS oligomerization and IRF3 activation (Jiang et al., 2016). This highlights again, that supramolecular signaling complexes can be prone to autoactivating mutations due to their ability to rapidly assemble into ordered signaling hubs, and the complexity with which they share common interactions and regulation.

As with the other supramolecular complexes discussed so far, a link with autophagy has been shown with CBM signalosomes too. Degradation of BCL10 in response to TCR stimulation occurs through a proteasome independent lysosomal dependent mechanism (Scharschmidt et al., 2004), suggesting autophagy as a likely route. As discussed later, the CBM complex also associates with p62 during TCR signaling in order to regulate the intensity of the signal (Paul et al., 2012).

## Mitochondria

Mitochondria act not only as the metabolic engine of the cell, but also as a key signaling hub filtering signals for cellular growth and energetics, innate immune and inflammatory signals, and also decisions to survive or die. The classical intrinsic mitochondrial apoptotic machinery comprised of Bax and Bak mediated pores regulated by BCL2 family pro and anti-apoptotic proteins is well characterized, if not completely understood (Edlich, 2018). As with the death domain family, the BCL2 family of proteins share common domains that they use for their interaction, namely the BCL2 Homology (BH) domain. Mechanistically, Bax and Bak form pores in the outer membrane of mitochondria upon an apoptotic stimulus. Contents of the mitochondrial intermembrane space then leak out, including cytochrome C and SMAC/DIABLO. Cytochrome C then binds APAF1 triggering the formation of the apoptosome thus activating caspase-9. Caspase-9 in turn activates effector caspases such as caspase-3 which carry out the end points of apoptosis (Figure 4). The BCL2 family proteins also form large complexes which are quite dynamic in their composition and function depending on the state of the cell, and may be antiapoptotic or proapoptotic [reviewed in Westphal et al. (2014), Cosentino and García-Sáez (2017), and Kale et al. (2018)]. As such they can be considered a kind of dynamic supramolecular complexes themselves that also use common domains (BH) to interact. In another link between mitochondria and inflammatory signaling, release of mitochondrial DNA (mtDNA) after Bak/Bak mediated permeabilization can also activate STING to trigger inflammation (McArthur et al., 2018; Riley et al., 2018).

The central role mitochondria play in regulating so many aspects of cellular homeostasis and immunity mean that they can in a sense be considered as a supramolecular signaling hub themselves. Given their importance it is not surprising then, that they are also heavily regulated and prone to quality control. Mitophagy is a specialized form of autophagy that causes the degradation of damaged mitochondria (Narendra et al., 2014). Mitophagy is analogous to aggrephagy in that targets on the mitochondria are ubiquitylated and cargo receptors such as p62 are recruited to the mitochondria followed by engulfment in autophagosomes and subsequent degradation in lysosomes (Figure 2, 3) (Geisler et al., 2010). Recent work by a number of labs has shown that mitophagy plays a role in regulating aspects of apoptosis and also associated inflammation. A recent study has shown the activation of Bax and Bak on mitochondria is associated with induction of autophagy, and that subsequently, apoptotic mitochondria are engulfed and degraded through autophagy (Lindqvist et al., 2017). Blockade of autophagy lead to enhanced production of interferon β (Lindqvist et al., 2017). Release of other mitochondrial contents in to the cytosol after outer membrane permeabilization can also be a stimulatory event, inducing cytokine production and DNA damage (Ichim et al., 2015; McArthur et al., 2018; Riley et al., 2018). BCL2 family of proteins integration into the outer membranes of mitochondria likely makes them a difficult target for direct action by specific degradation through autophagy, however, evidence exists for a role of Parkin (E3 ubiquitin ligase responsible for ubiquitylating damaged mitochondria during mitophagy) in regulating BCL2 complexes as well as mitophagy. Parkin can ubiquitylate MCL-1 leading to its degradation and cell death in response to mitochondrial damaging agents such as valinomycin (Zhang et al., 2014). Less damaging treatments such as cccp are reported to induce mitophagy instead (Zhang et al., 2014). Loss of mitophagy may contribute to inflammation in a number of ways including accumulation of ROS, release of mtDNA from damaged mitochondria as well as persistence of signaling platforms such as MAVS that are localized on the mitochondrial membrane.

### TBK1 as a Central Regulator

An interesting connection between the activation of these receptor signaling complexes and their degradation by autophagy is the activation of the Tank Binding Kinase 1 (TBK1). TBK1 is required for IRF activation from many of the receptors complexes described in this review including TLR3/4 via TRIF, STING and MAVS. TBK1 is also a well characterized activator of autophagic cargo receptors including p62, optineurin and NDP52 (Pilli et al., 2012; Korac et al., 2013; Matsumoto et al., 2015; Yang et al., 2016; Oakes et al., 2017; Cho et al., 2018). TBK1 phosphorylates these receptors, enhancing their recruitment of the autophagic machinery and therefore promoting degradation of the target complexes (Figure 2). This was shown directly for STING, but also for mitochondria and bacteria. Recently TBK1 was also identified as being recruited to TNFR1 where it does not activate IRF3, but instead played an essential role in regulating survival (Figure 3). Loss of TBK1 expression leads to RIPK1 dependent apoptosis and reduced levels can replicate ALS in mice (Lafont et al., 2018; Xu et al., 2018). This was shown to be dependent on TBK1 mediated phosphorylation of RIPK1. Given the recent observations about TNFR1 and TBK1 mediated survival, it is possible that TBK1 activation may also promote the degradation of RIPK1 complexes in reducing the amount of activated complex-II in response to TNF, however, this remains to be shown. Of note is that TBK1 is also a critical component of enhanced autophagy and NF-κB activation in K-Ras-dependent non-small cell lung carcinoma (NSCLC) (Newman et al., 2012). The role of TBK1 in activating autophagy receptors and its recruitment to and activation by supramolecular complexes described here is indicative of the importance of this pathway in dampening inflammatory and cell death signals.

## Autophagy Receptors Acting as Signaling Scaffolds Too

In another twist to the role of autophagy in regulating supramolecular signaling complexes, some cargo receptors such as p62 can promote signaling and assembly of the complexes prior to their degradation (Figure 2B). This has been reported for caspase-8 activation upon TRAIL stimulation (Jin et al., 2009), whereby p62 promoted the aggregation of caspase-8 in a cullin-3 dependent fashion, thus suggesting that aggregation by p62 enhanced the signal triggering apoptosis, but at the same time lead to the ultimate degradation of the complex and its silencing.

Recently a role for HDAC6 has been shown in clearance of *Listeria monocytogenes* (Moreno-Gonzalo et al., 2017). In this context loss of HDAC6 in DCs resulted in enhanced bacterial load, due to defects in autophagy, and also strongly reduced activation of NF-κB and MAPK pathways. It was proposed that by interacting with MYD88 that HDAC6 promoted its aggregation and activation of downstream signaling pathways. While no specific mechanism for this is given, HDAC6 may promote association of MYD88 with autophagy receptors such as p62 to enhance its activity before being degraded. Additionally loss of p62 was shown to reduce cytokine production, NF-κB and ERK activation in response to TLR2 and TLR6 activation in keratinocytes (Lee et al., 2011). In a similar fashion to the above examples, p62 promoted NF-κB activation prior to degradation of BCL10 in TCR signaling (Paul et al., 2012, 2014), however, BCl10 degradation ultimately silences NF-κB activation (Scharschmidt et al., 2004). NOD2 also shows reduced NF-κB activation in response to ligand in the absence of p62 (Park et al., 2013). Of note is that is required for TRAF6 dependent ubiquitylation of NEMO/IKKγ, and loss of p62 blocks IL-1β induced NF-κB substantially (Zotti et al., 2014). Additionally p62 is required for RAS induced NF-κB in cancer through TRAF6 ubiquitylation and IKK activation (Durán et al., 2008). Together these data support the idea that these large signaling complexes that become ubiquitylated also use this aggregation phase to enhance signaling prior to silencing. While p62 is by far the most studied of the autophagy cargo receptors, it is likely that there is some redundancy and that the other cargo receptors also exhibit signal amplifying activities prior to their degradation. Thinking of these adapters as cargo receptors may actually be too simplistic for their role in signal regulation, and instead perhaps they should be thought of more as generalized modulators or scaffolds for tuning signal strength and duration.

### Loss of Autophagy in Various Diseases Associated With Inflammation and Cell Death

A number of diseases are associated with deficiencies in autophagy, many of which are inflammatory in nature and in a number of cases show direct links to proteins from supramolecular signaling complexes involved in cell death and inflammatory signaling. Gaucher’s Disease is a lipid storage disease caused by mutations in glucocerebrosidase that results in accumulation of the sphingolipid glucocerebroside in lysosomes, effectively blocking their function. Thus, as a byproduct, the autophagy pathway is also backed-up and blocked by a failure to degrade targets in the lysosome (Settembre et al., 2008). Gaucher’s disease is associated with a strong hyperinflammation and splenomegaly and interestingly, in mouse models, it was shown that it could be largely blocked by loss of RIPK3, suggesting a potential role for RIPK3 mediated cell death as a possible driver of the disease (Vitner et al., 2014). While it has yet to be shown, it is intriguing to speculate that active RIPK3, assembled into fibrillar complexes through the RHIM domain do not get degraded, and then promote either cell death or inflammation directly. Indeed increased RIPK3 levels are seen in Gaucher’s patients (Vitner et al., 2014).

Niemann Pick disease is another lysosomal disease that is associated with inflammatory pathology, particularly Crohn’s disease like symptoms. Niemann Pick diseases are caused by failure to metabolize Sphingomyelin for various reasons, leading to lysosomal disfunction (Guo et al., 2016). While it has not directly been shown that Niemann Pick is regulated by RIPK3 in a similar fashion to Gaucher’s disease, the possibility remains. As mentioned, a particular pathology associated with Niemann-Pick is the development of Crohn’s Disease like pathology. This was reported to be associated with decreased xenophagy in a manner similar to loss of function of two other well-known Crohn’s Disease associated proteins, Nod2 and XIAP (Schwerd et al., 2017), both of which also positively regulate autophagy (Homer et al., 2010; Gradzka et al., 2018). Mutations in NOD2 lead to loss of NF-κB activation, as do many of the mutations in XIAP that are associated with disease, suggesting that failure to activate this pathway is the initial cause of the pathology, however, recent studies have shown that NOD2 and XIAP both also have roles in targeting invasive bacteria for xenophagy (Homer et al., 2010; Gradzka et al., 2018). How this may be coordinated is not clear, but the association of other autophagy related mutations such as the Atg16L1, NDP52, Optineurin, and others CD suggest that specific defects in the autophagy process may be a key trigger as well. At least part of the inflammation seen may be associated with a concomitant failure to silence assembled signaling complexes within the usual time frame. Additionally, there are a number of neuronal diseases associated with defects in autophagy that may also have an inflammatory element. These include Alzheimer’s disease and Parkinson’s disease to give just two prominent examples. Again, a role for degradation of signaling complexes may also play a significant role in the inflammation and cell death seen in these diseases.

## Conclusion

Formation of large supramolecular complexes as signaling hubs is emerging as a unifying theme in signaling and it is likely that many more receptor complexes will demonstrate this capacity. The fact that so many receptors all share common structural domains and signaling targets supports this and highlights the crosstalk that many of these receptors have in regulating the strength, and severity of signals that are produced. The propensity of these complexes to assemble is also highlighted by the numerous, although rare, genetic immune diseases associated with auto-activation of components of the complexes discussed here. The common theme of induction of autophagy, recruitment to cargo receptors to amplify signals prior to their degradation provides a tightly regulatable circuit for negative regulation of these important signaling complexes and suggest that autophagy induction may provide a useful target in helping to prevent some of these diseases at their source.

## Author Contributions

IG conceived of and wrote the manuscript.

## Conflict of Interest Statement

The authors declare that the research was conducted in the absence of any commercial or financial relationships that could be construed as a potential conflict of interest.
